# Occurrence of *bla*_DHA-1_ mediated cephalosporin resistance in *Escherichia coli* and their transcriptional response against cephalosporin stress: a report from India

**DOI:** 10.1186/s12941-017-0189-x

**Published:** 2017-03-21

**Authors:** Birson Ingti, Deepjyoti Paul, Anand Prakash Maurya, Debajyoti Bora, Debadatta Dhar Chanda, Atanu Chakravarty, Amitabha Bhattacharjee

**Affiliations:** 1 Department of Microbiology, Assam University, Silchar, 788011 India; 2 Department of Microbiology, Silchar Medical College and Hospital, Silchar, 788014 India; 3 Department of Statistics, Dibrugarh University, Dibrugarh, India

## Abstract

**Background:**

Treatment alternatives for DHA-1 harboring strains are challenging as it confers resistance to broad spectrum cephalosporins and may further limit treatment option when expressed at higher levels. Therefore, this study was designed to know the prevalence of DHA genes and analyse the transcription level of DHA-1 against different β-lactam stress.

**Methods:**

Screening of AmpC β-lactamase phenotypically by modified three dimensional extract method followed by Antimicrobial Susceptibility and MIC determination. Genotyping screening of β-lactamase genes was performed by PCR assay followed by their sequencing. The *bla*_DHA-1_ transcriptional response was evaluated under different cephalosporin stress by RT PCR. Transferability of *bla*_DHA_ gene was performed by transformation and conjugation and plasmid incompatibility typing, DNA fingerprinting by enterobacterial repetitive intergenic consensus sequences PCR.

**Results:**

16 DHA-1 genes were screened positive from 176 *Escherichia coli* isolates and primer extension analysis showed a significant increase in DHA-1 mRNA transcription in response to cefotaxime at 8 µg/ml (6.99 × 10^2^ fold), ceftriaxone at 2 µg/ml (2.63 × 10^3^ fold), ceftazidime at 8 µg/ml (7.06 × 10^3^ fold) and cefoxitin at 4 µg/ml (3.60 × 10^4^ fold) when compared with untreated strain. These transcription data were found significant when analyzed statistically using one way ANOVA. Four different ESBL genes were detected in 10 isolates which include CTX-M (n = 6), SHV (n = 4), TEM (n = 3) and OXA-10 (n = 1), whereas, carbapenemase gene (NDM) was detected only in one isolate. Other plasmid mediated AmpC β-lactamases CIT (n = 9), EBC (n = 2) were detected in nine isolates. All DHA-1 genes detected were encoded in plasmid and incompatibility typing from the transformants indicated that the plasmid encoding *bla*_DHA-1_ was carried mostly by the FIA and L/M Inc group.

**Conclusion:**

This study demonstrates the prevalence of DHA-1 gene in this region and highlights high transcription of DHA-1 when induced with different β-lactam antibiotics. Therefore, cephalosporin treatment must be restricted for the patients infected with pathogen expressing this resistance determinant.

## Background

*Escherichia coli (E. coli)* possess a chromosomal cephalosporinase gene, which is regulated by a weak promoter and a transcriptional attenuator. The gene confers resistance only to narrow-spectrum cephalosporins [1, 2]. However, spontaneous mutations in the promoter, as well as transcriptional attenuator region of the AmpC gene may induce constitutive overproduction of the cephalosporinase resulting in resistance to penicillins and broad-spectrum cephalosporins (e.g. cefotaxime, ceftazidime, ceftriaxone, aztreonam etc.) [3, 4]. Besides hyper-production of the chromosomally encoded enzyme, the presence of one or more plasmid-mediated AmpC β-lactamases along with other intrinsic mechanisms in *E. coli* leads to resistance against multiple antimicrobial agents, compromising the efficacy of treatment [5–7]. Six families of plasmid-encoded AmpC β-lactamases were described based on their sequence similarities as CIT, FOX, MOX, DHA, EBC, and ACC [8]. The most commonly recognized plasmid-mediated AmpC among the strains of *E. coli* includes the CMY-2 type which belongs to the CIT family [9, 10].

DHA-1, another plasmid-mediated AmpC β-lactamase, belonging to DHA family was found increasingly among Enterobacteriaceae in many parts of the world and was a growing concerned in the medical world as it leads to treatment failure [7]. It was first characterized in a *Salmonella enteritidis* which has the ability to hydrolyze penicillins, cephamycin, including broad spectrum cephalosporin leaving physicians with limited antibiotic choices. It was also the first plasmid-encoded β-lactamase found to be inducible and can be expressed in high levels [11, 12]. So far a total of 24 gene types of DHA family have been reported (http://www.ncbi.nlm.nih.gov/projects/pathogens/submit_beta_lactamase). The regulation of this β-lactamase expression is closely linked to cell wall recycling and involves at least three genes: *ampR* (codes for a transcriptional regulator of the LysR family), *ampG* (codes for a transmembrane permease) and *ampD* (codes for a cytosolic *N*-acetyl-anhydromuramyl- l-alanine amidase) [13].

Though it was well established that β-lactam antibiotics are potent inducers of class C in most of the members of the family Enterobacteriaceae [7], there is no relevant information on the level of AmpC expression taking place when the strains with incomplete regulatory elements were under antibiotic stress. Therefore, this study was undertaken to investigate the transcriptional response of DHA-1 under various cephalosporin’s stresses.

## Methods

### Bacterial strains

A total of 176 consecutive, non-duplicate *Escherichia* isolates were collected from different clinical specimens (mostly from urine followed by pus) obtained from different Wards/OPD of Silchar Medical College and Hospital, India from October 2012 to March 2013. The isolates were identified by cultural characteristics, biochemical reactions and further confirmed by 16S rDNA sequencing using primers, a forward primer 5′-AGAGTTTGATCMTGGCTCAG-3′ and a reverse primer 5′-TACGGYTACCTTGTTACGACTT-3′.

### Screening of AmpC β-lactamase by cefoxitin disc test and modified three dimensional extract method

Preliminary screening of AmpC β-lactamase was carried out on Mueller–Hinton Agar plates containing cefoxitin (30 µg) (Hi Media, Mumbai). Isolates with inhibition zones of less than 18 mm, were considered as screen positives [14]. The suspected AmpC β-lactamase producers were further confirmed by modified three dimensional extract test (M3DET) [15]. *Escherichia coli* ATCC 25922 and *Enterobacter cloacae* P99 were used as negative and positive control respectively.

### Antimicrobial susceptibility and minimum inhibitory concentrations (MIC’s) determination

Antimicrobial susceptibility was determined by Kirby Bauer disc diffusion method on Mueller–Hinton Agar plates. Following antibiotics were used: amikacin (30 μg), gentamicin (10 μg), ciprofloxacin (30 μg), trimethoprim/sulphamethoxazole (1.25/23.75 μg), tigecycline (15 μg) (Hi Media, Mumbai). MIC’s of various antibiotics were also determined on Mueller–Hinton Agar plates by agar dilution method according to CLSI and EUCAST guidelines [16, 17]. Following antibiotics were used: cefotaxime, ceftazidime, ceftriaxone, cefepime, imipenem, meropenem, ertapenem and aztreonam (Hi-Media, Mumbai, India).

### Detection of DHA gene by polymerase chain reaction

Polymerase chain reaction (PCR) was performed targeting all the DHA genes by using a pair of primers as listed in Table 1. Isolates positive for DHA genes were further investigated for the presence of other AmpC gene families, namely: CIT, ACC, FOX and EBC [18]. PCR amplification was performed using 30 µl of total reaction volume. Reactions were run under the following conditions: initial denaturation at 95 °C for 2 min, 34 cycles of 95 °C for 15 s, 51 °C for 1 min, 72 °C for 1 min and final extension at 72 °C for 7 min.

**Table 1:** List of oligonucleotide primers for amplification of β-lactamase genes

Serial no.	Targets	Primers pairs	Sequence (5′→3′)	Product size (bp)	Reference
1	DHA-1 and DHA-2	DHA F DHA R	TGATGGCACAGCAGGATATTC GCTTTGACTCTTTCGGTATTCG	997	[18]
2	KPC	KPC F KPC R	5′-CATTCAAGGGCTTTCTTGCTGC-3′ 5′-ACGACGGCATAGTCATTTGC-3′	538	[20]
3	IMI/NMC	IMI/NMC F IMI/NMC R	5′-CCATTCACCCATCACAAC-3′ 5′-CTACCGCATAATCATTTGC-3′	440	[21]
4	SME	SME F SME R	5′-AACGGCTTCATTTTTGTTTAG-3′ 5′-GCTTCCGCAATAGTTTTATCA-3′	831	[22]
5	VIM	VIM F VIMR	5′-GATGGTGTTTGGTCGCATA-3′ 5′-CGAATGCGCAGCACCAG-3′	390	[23]
6	IMP	IMP F IMP R	5′-TTGACACTCCATTTACDG-3′ 5′-GATYGAGAATTAAGCCACYCT-3′	139	[23]
7	NDM	NDM F NDM R	5′-GGGCAGTCGCTTCCAACGGT-3′ 5′-GTAGTGCTCAGTGTCGGCAT-3′	476	[24]
8	DHA-1	DHA-RT F DHA-RT R	5′-TGATGGCACAGCAGGATATTC-3′ 5′-TACTTACAGATCCGAGCTCAA-3′	144	This study

PCR products were purified by QIAquick Gel Extraction Kit (QIAGEN, Germany) and sequenced. Sequence results were analysed using a BLAST suite program of NCBI (http://blast.ncbi.nlm.nih.gov/Blast.cgi).

### Molecular characterization of ESBL and carbapenemase genes by multiplex PCR

For amplification and characterization of ESBL genes, a set of five primers were used, namely: TEM, CTX-M, SHV, OXA-2, and PER [19]. Reactions were run under the following conditions: initial denaturation at 94 °C for 5 min, 33 cycles of 94 °C for 35 s., 51 °C for 1 min, 72 °C for 1 min and a final extension at 72 °C for 7 min.

For amplification and characterization of carbapenemase genes, a set of seven primers were used, namely: KPC, IMI, NMC, SME, VIM, IMP, and NDM (Table 1). Reactions were run as described previously.

### Transcriptional expression analysis of *bla*_DHA-1_ by quantitative realtime PCR

Expression of the *bla*_DHA-1_ gene was studied in response to cefoxitin, cefotaxime, ceftriaxone and ceftazidime stress at different concentrations (2, 4, 8 µg/ml) and was determined by inoculating the organisms harboring *bla*_DHA-1_ in Luria–Bertani broth (Hi-media, Mumbai, India). Isolate without any antibiotic pressure was used as a control. A total RNA was isolated using Qiagen RNase Mini Kit (Qiagen, Germany), immediately reverse transcribed into cDNA by using QuantiTect^®^ reverse transcription kit (Qiagen, Germany). The cDNA was quantified by Picodrop (Pico 200, Cambridge, UK) and quantitative real time PCR was performed using Power Sybr Green Master Mix (Applied Biosystem, Warrington, UK) in step one plus real time detection system (Applied Biosystem, USA). The house keeping gene *rpsel* of *E. coli* was used as an internal standard [25]. DHA-1 positive isolates showing resistance to broad spectrum cephalosporins and also devoid of other β-lactamases was selected for this study. The primer used for amplification of DHA-1 is listed in Table 1. PCR reactions were performed in triplicates for the isolate. The reaction was run under the following conditions: 95 °C for 2 min, 32 cycles of 95 °C for 20 s, 48 °C for 40 s and 72 °C for 1 min. The relative expression of *bla*_DHA-1_ at a different antibiotics pressure was determined by the ΔΔC_t_ method. Relative quantification was compared with strain grown for 16 h without any antibiotic pressure.

### Statistical analysis

The changes in DHA-1 mRNA expression in response to different β-lactam antibiotic stresses at different concentration were analyzed using one-way ANOVA followed by Tukey–Kramer (Tukey’s W) multiple comparison test using SPSS version 17.0. Differences were considered statistically significant at both 5 and 1% level when p < 0.05. Data are presented as mean fold change + standard error of the mean.

### Plasmid preparation

The bacterial isolates were cultured in Luria–Bertani broth (LB broth) containing 0.25 µg/ml of cefotaxime. Cultures were incubated on shaker incubator overnight at 37 °C, 160 rpm. Plasmids were purified by QIA prep Spin Miniprep Kit (QIAGEN, Germany).

### Transferability of *bla*_DHA_ gene by transformation and conjugation

The transformation experiments were carried out by heat shock method [26] using *E. coli* DH5α as the recipient. Transformants were selected on cefotaxime (0.5 µg/ml) containing LB Agar plates.

Conjugation experiments were carried out between clinical isolates as donors and a streptomycin resistant *E. coli* strain B (Genei, Bangalore) as the recipient. An overnight culture of the bacteria was diluted in Luria–Bertani broth (Hi-Media, Mumbai, India) and was grown at 37 °C till the O.D. of the recipient and donor culture reached 0.8–0.9 at A_600_. Donor and recipient cells were mixed at 1:5 donor-to-recipient ratios and transconjugants were selected L.B Agar plates supplemented with cefotaxime (0.5 µg/ml) and streptomycin (600 µg/ml).

### Plasmid incompatibiltiy typing

For detection of incompatibility group type of plasmid carrying *bla*_DHA_, PCR based replicon typing was carried out, targeting 18 different replicon types, to perform five multiplex and three simplex PCRs to amplify the FIA, FIB, FIC, HI1, HI2, I1-Ig, L/M, N, P, W, T, A/C, K, B/O, X, Y, F and FIIA replicons [27].

### DNA fingerprinting by enterobacterial repetitive intergenic consensus sequences PCR

Typing of all *bla*_DHA-1_ producing *E. coli* isolates was done by enterobacterial repetitive intergenic consensus (ERIC) PCR as described previously [28]. Isolates were put into cluster based on banding pattern and dendogram was prepared by NTSYS software.

## Results

**Fig. 1:**
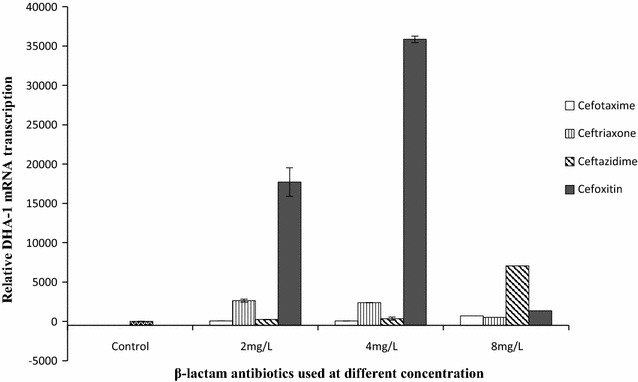
Transcriptional analysis of DHA-1 espression by RT-PCR. Total bacterial RNA was isolated from mid-log- phase cultures of *E. coli*. The *error bars* represent the standard deviations of the means of triplicate samples

During the study period, a total of 176 *E. coli* isolates were obtained from different clinical samples. Among these, 110 (62.5%) were resistant to cefoxitin and 63 (35.8%) isolates were found to show AmpC activity by M3DET. By performing PCR, 16 isolates were detected for DHA genes and showed a sequence identical to that of DHA-1 (Table 1). These isolates harboring DHA-1 gene were selected for further study. Among DHA-1 positive isolates four different ESBL genes were detected in 10 isolates which include CTX-M (n = 6), SHV (n = 4), TEM (n = 3) and OXA-10 (n = 1). Carbapenemase gene (NDM-1) was detected only in one isolate. Other plasmid mediated AmpC β-lactamase CIT (n = 9), EBC (n = 2) were detected in nine isolates that carried either CTX-M (n = 3),SHV (n = 1), TEM (n = 1), NDM (n = 1) alone or CTX-M plus SHV(n = 2), CTX-M plus TEM (n = 1) and OXA-10 plus SHV (n = 1) (Table 2). These isolates harboring AmpC β-lactamase were mostly obtained from Surgery and medicine ward. To demonstrate whether DHA-1 expression would take place in the presence of different cephalosporins at a different concentration, an *E.coli* strain BM-567 (Table 2) harboring only DHA-1 β-lactamase and showing resistance to broad spectrum cephalosporins was selected. The fold increase in mRNA production was measured using primer extension analysis. It was observed that there was a significant increase in the expression of DHA-1 gene in response to cefotaxime, ceftriaxone, ceftazidime but not as high as those for cefoxitin when compared with the basal level without antibiotic pressure (Fig. 1). Though increased in transcription was observed in response to these β-lactam antibiotics, high transcript level were achieved when induced by cefotaxime at 8 µg/ml (6.99 × 10^2^ fold), ceftriaxone at 2 µg/ml (2.63 × 10^3^ fold), ceftazidime at 8 µg/ml (7.06 × 10^3^ fold) and cefoxitin at 4 µg/ml (3.60 × 10^4^ fold) (Fig. 1). The ANOVA and Tukey–Kramer (Tukey’s W) multiple comparison test for checking the differences in the expression of DHA-1 was found to be significant (*p* value is less than 0.05; Table 3).

**Table 2:** Clinical history, their molecular details and resistance profile of DHA-1 gene-positive *E. coli* isolates

Sl. No.	Sample ID	Age (years)	Sex	Ward/clinics	Type of clinical specimen	^a^ESBL genes detected	Carbapenemase genes detected	Other plasmid AmpC genes	(Inc type)	Resistance profile	MIC of β-lactam (mg/l)							
											CTX	CAZ	CRO	FEP	ATM	IMP	MEM	ETP
1	BM12	35	Male	Surgery	Pus	–	–	–	K	AMK, GEN, SXT	>512	>512	256	>512	64	16	8	8
2	BM26	107	Female	Pediatrics	Urine	TEM	–	–	FIA,	CIP, AMK, GEN, SXT	64	128	64	8	64	<2	<2	<2
3	BM59	55	Female	Medicine	Urine	–	–	–	FIA	CIP, AMK, GEN, SXT	128	64	64	16	128	<2	<2	<2
4	BM63	60	Female	Surgery	Pus	CTX-M	–	CIT	–	CIP, AMK, GEN, SXT	128	128	256	16	64	<2	<2	<2
5	BM130	27	Female	Surgery	Pus	TEM	–	CIT, EBC	HI1, L/M	CIP, AMK, GEN, SXT	>512	512	>512	64	256	16	4	4
6	BM138	45	Male	Surgery	Pus	CTX-M	–	–	–	CIP, GEN, SXT	64	128	128	32	128	<2	<2	<2
7	BM197	30	Female	Surgery	Pus	–	–	CIT	L/M	CIP, AMK, GEN, SXT	512	>512	256	64	512	2	<2	<2
8	BM230	43	Female	Surgery	Pus	CTX-M, SHV	–	CIT	L/M	CIP, AMK, GEN, SXT	64	128	256	32	256	16	8	16
9	BM252	7	Female	Paediatrics	Urine	SHV	–	CIT, EBC	F1B, FIA	CIP, AMK, GEN, SXT	>512	>512	>512	128	256	8	<2	<2
10	BM355	10	Male	Paediatrics	Urine	–	NDM	CIT	FIA	CIP, AMK, SXT	>512	512	>512	8	256	<2	<2	<2
11	BM409	61	Male	Medicine	Urine	CTX-M	–	CIT	K	CIP, AMK, GEN, SXT	64	64	32	16	128	<2	<2	<2
12	BM441	40	Male	Medicine	Stool	CTX-M, SHV	–	–	FIA	CIP, AMK, GEN, SXT	32	64	128	32	256	<2	<2	<2
13	BM508	48	Male	Surgery	Pus	–	–	CIT	I1	CIP, AMK, GEN	32	32	32	16	256	<2	<2	<2
14	BM520	55	Female	Surgery	Pus	OXA-10, SHV	–	–	–	CIP, GEN, SXT	16	128	128	32	32	<2	<2	<2
15	BM567	30	Male	Medicine	Urine	–	–	–	–	CIP, AMK, GEN, SXT	128	256	256	32	256	<2	<2	<2
16	BM576	40	Female	Medicine	Urine	CTX-M, TEM	–	CIT	K, B/O	AMK, GEN, SXT	>512	>512	>512	128	>512	16	16	32

*AMK* amikacin; *GEN* gentamycin; *CIP* ciprofloxacin; *SXT* cottrimoxazole; *CTX* cefotaxime; *CAZ* ceftazidime; *CRO* ceftriaxone; *FEP* cefepime; *ATM* aztreonam; *IMP* imipenem; *MEM* meropenem; *ETP* ertapenem

^a^Extended spectrum β-lactamase

**Table 3:** Statistical analysis of changes in DHA-1 mRNA expression in response to different-lactam antibiotic stress at different concentration using one way ANOVA

Sl no.	β-lactam antibiotics	Value (Mean ± SEM)				p value
		NA	2 mg/l	4 mg/l	8 mg/l	
1	Cefotaxime	1.00 ± 0	56.66 ± 1.18	58.10 ± 0.58	699.25 ± 5.27	0.001^*^
2	Ceftriaxone	1.00 ± 0	2634.69 ± 16.58	2383.97 ± 20.73	510.70 ± 6.04	
3	Ceftazidime	1.00 ± 0	249.50 ± 11.63	356.27 ± 14.20	7059.48 ± 60.91	
4	Cefoxitin	1.00 ± 0	17721.31 ± 608.47	35855.21 ± 1050.38	1363.69 ± 237.37	

*NA* no antibiotic; *SEM* standard error of the mean

^*^Significant (p < 0.05)

**Fig. 2:**
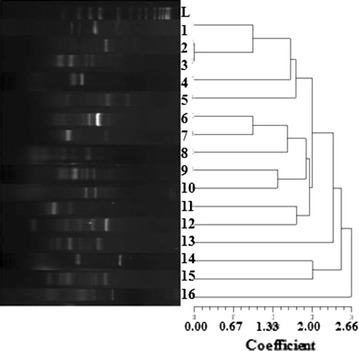
ERIC PCR analysis of *E. coli* producing DHA-1 β-lactamase.* Lane *L: molecular weight marker;* lanes* 1–16: isolates harboring DHA-1 β-lactamase

Typing by ERIC-PCR confirmed 16 different haplotypes (Fig. 2) indicating the diversity of the isolates. The susceptibility pattern of these *bla*_DHA-1_ harboring isolates showed resistance towards β-lactam including broad spectrum cephalosporin but most of them were susceptible against a carbapenem group of drugs. They also show susceptibility to tigecycline and moderate to high resistance against amikacin, gentamycin, co-trimoxazole, ciprofloxacin. The MICs of selected β-lactam antibiotics for all the parental strains harboring DHA-1 were found to be above breakpoint level (Table 2). The transformation experiment could establish that DHA-1 was encoded in plasmid however, conjugation experiment revealed that only 4 isolates could conjugatively transfer DHA-1 gene in *E. coli* strain B which was confirmed by PCR analysis. On performing incompatibility typing it was established that most of the transformants with DHA-1 were associated with K, FIA, L/M, FIB, HI1, B/O & I1 *Inc* group (Table 2).

## Discussion

The first plasmid mediated AmpC β-lactamase, to be reported was CMY-1, in 1989 [29]. Since then, several plasmid-encoded AmpC β-lactamases (ACC, FOX, MOX, CMY, ACT, etc.) have been reported in several genera of bacteria, including *Salmonella* spp., *Pseudomonas* spp., *Proteus mirabilis* and *Klebsiella pneumoniae* [7]. Among them plasmid encoded DHA-1; a clinically important AmpC β-lactamase was the first β-lactamase found to be inducible and can be expressed at higher levels in strains having AmpR regulatory gene [11, 30]. This plasmid- mediated β-lactamase is now being increasingly detected in a strain of *E. coli* worldwide [31–33] and early detection of this β-lactamase (DHA-1) is mandatory for better antibiotic therapy and also to prevent further spread. The present study reports the prevalence of DHA-1 (9%) among *E. coli* strains in this region which is quite high compared to other studies [30, 32, 33] and typing of these DHA-1 harboring isolates by ERIC PCR revealed diverse haplotypes, indicating the spread of the DHA-1 gene through horizontal transfer. Based on the present susceptibility data (Table 2) and previous studies [11, 12], carbapenem and other non-β-lactam antibiotics such as tigecycline could be better drugs of choice for the treatment of infections caused by *E. coli* producing DHA-1.

From the earlier study, it appears that *E. coli* lack one of the regulatory component (AmpR gene), which leads to the lower level, non-inducible expression of AmpC [34]. However, inducible cephalosporinase (*bla*_CMY-13_) found associated with an AmpR gene was detected recently in a strain of *E. coli* [35]. Several broad spectrum cephalosporins were believed to increase the expression of AmpC β-lactamase [36], although the concentration which leads to increase in the expression of AmpC β-lactamase was not established. This study demonstrates higher transcription of DHA-1 when induced with different cephalosporins. These differences in the relative amounts of RNA transcription of DHA-1 gene, when induced with different cephalosporins at a concentration below MIC level suggest that the transcription varies depending on the level of antibiotics stress. Higher AmpC production was supported by another finding, where *bla*MIR-1, a plasmid-encoded *AmpC* gene exhibited a 95-fold increase in expression relative to WT *AmpC* [37]. Concentration dependent expression of AmpC cephalosporinase was also observed in a strain of *Pseudomonas aeruginosa*, when the strain was induced with cefoxitin or clavulanic acid at 8, 16 and 50 µg/ml [38]. So far, the factors behind the quantitative differences of AmpC expression in *E.coli* strain when exposed to different β-lactam concentration is unknown. A transformation experiment could establish that all the DHA-1 gene were encoded in a plasmid which is in agreement with the previous study [12, 30–33] and Incompatibility typing from the transformants indicated that the plasmid encoding *bla*_DHA-1_ was carried mostly by FIA and L/M Inc group as found in another study [39]. Although detection of other Inc group, namely HI1, FIB, I1, K in the present study was mostly associated with CMY-2 and ACC harboring strains [39]. Plasmids carrying genes for AmpC β-lactamases often carry ESBL genes such as CTX-M [40, 41] as found in the present study, where most of these DHA-1 harboring isolates co-harbour ESBL genes (Table 2). Co-existence of New Delhi metallo-β-lactamase (NDM) gene was also observed in one isolate as the high prevalence of the *E. coli* harboring a metallo-β-lactamase known as the NDM has been increasingly observed in the Indian subcontinent [42].

## Conclusion

Strains harboring plasmid mediated AmpC (DHA-1) genes are often resistant to multiple antimicrobial agents and the overexpression of this resistant determinant when induced with different cephalosporins stress will further limit treatment option. The present study demonstrates that higher expression of DHA-1 takes place when induced with specific concentrations of β-lactam antibiotics, although further research is required to understand the factors behind the upregulation of DHA-1 gene in the future. Therefore, revision in cephalosporin usage policy is required for effective treatment of patients infected with pathogen harboring this mechanism.
